# Comparisons of the prediction models for undiagnosed diabetes between machine learning versus traditional statistical methods

**DOI:** 10.1038/s41598-023-40170-0

**Published:** 2023-08-11

**Authors:** Seong Gyu Choi, Minsuk Oh, Dong–Hyuk Park, Byeongchan Lee, Yong-ho Lee, Sun Ha Jee, Justin Y. Jeon

**Affiliations:** 1 Department of Sports Industry Studies, Yonsei University, Seoul, Republic of Korea; 2 Frontier Research Institute of Convergence Sports Science, Yonsei University, Seoul, Republic of Korea; 3 Gauss Labs, Seoul, Republic of Korea; 4 Department of Internal Medicine, Yonsei University College of Medicine, Seoul, Republic of Korea; 5 Institute for Health Promotion, Graduate School of Public Health, Yonsei University, Seoul, Republic of Korea; 6 Exercise Medicine Center for Diabetes and Cancer Patients, ICONS, Seoul, Republic of Korea; 7 Cancer Prevention Center Shinchon Severance, Yonsei University College of Medicine, Shinchon-Dong, Seodaemun-Gu, Seoul, 120-749 Republic of Korea

**Keywords:** Diseases, Health care, Medical research

## Abstract

We compared the prediction performance of machine learning-based undiagnosed diabetes prediction models with that of traditional statistics-based prediction models. We used the 2014–2020 Korean National Health and Nutrition Examination Survey (KNHANES) (N = 32,827). The KNHANES 2014–2018 data were used as training and internal validation sets and the 2019–2020 data as external validation sets. The receiver operating characteristic curve area under the curve (AUC) was used to compare the prediction performance of the machine learning-based and the traditional statistics-based prediction models. Using sex, age, resting heart rate, and waist circumference as features, the machine learning-based model showed a higher AUC (0.788 vs. 0.740) than that of the traditional statistical-based prediction model. Using sex, age, waist circumference, family history of diabetes, hypertension, alcohol consumption, and smoking status as features, the machine learning-based prediction model showed a higher AUC (0.802 vs. 0.759) than the traditional statistical-based prediction model. The machine learning-based prediction model using features for maximum prediction performance showed a higher AUC (0.819 vs. 0.765) than the traditional statistical-based prediction model. Machine learning-based prediction models using anthropometric and lifestyle measurements may outperform the traditional statistics-based prediction models in predicting undiagnosed diabetes.

## Introduction

The Diabetes Fact Sheet in Korea 2020 from the Korean Diabetes Association reported that the prevalence of type 2 diabetes (hereafter “diabetes”) in Korean adults aged ≥ 30 years in 2018 was 13.8% (approximately 4.9 million)^1^. However, detecting diabetes is challenging, given the asymptomatic state at an early stage of diabetes. Consequently, many cases of diabetes are not diagnosed until after one’s diabetes complications have deteriorated^2^, and the optimal timing of diabetes treatment is often delayed^3,4^.

Therefore, it is imperative to identify an easy and accessible diabetes prediction at an early stage to effectively treat and manage diabetes and prevent its complications. Growing evidence has suggested some diabetes prediction models using “non-invasive” data, including sociodemographic, clinical, and key health characteristics (e.g., age, waist circumference [WC], family history of diabetes, smoking status, alcohol consumption, and resting heart rate [RHR])^5,6^. Based on the magnitude of the relationships between candidate diabetes risk factors and diabetes, there are some (early stage) diabetes prediction models using either a self-report survey using the diabetes risk score (DRS)^7,8^ or applying various algorithms from a machine learning perspective^9,10^.

In Korea, diabetes prediction models have been established using data from the Korean National Health and Nutrition Examination Survey (KNHANES) and the Korean Genome and Epidemiology Study. However, the previous Korean diabetes prediction models were limited by (1) insufficiently high (i.e., 0.74 to 0.765) the receiver operating characteristic curve area under the curve (AUC) in the models^5,6^ and (2) low accessibility, given that the models used blood lipid profiles (e.g., fasting glucose, glycated hemoglobin [HbA1c], triglyceride, and total cholesterol)^11,12^. Furthermore, Jang et al. suggested a previous diabetes prediction model^13^ may be valid in a specific condition only when adjusting for the proportion of diabetic vs. non-diabetic individuals at 1:1. Additionally, some research was at high risk of “overfitting” because the external validity of models was not examined given that the “training and internal validation set” and “external validation set” were not properly differentiated^14^.

To fill the knowledge gap in the literature, the objective of this study was to compare the performance of machine learning (ML)-based prediction models and traditional statistics (TS)-based prediction models using non-invasive, highly accessible clinical variables (e.g., age, sex, anthropometry, family history of diabetes, lifestyle behaviors). We hypothesized that the prediction performance of the ML-based undiagnosed diabetes prediction models would be superior to that of the TS-based undiagnosed diabetes prediction models.

## Methods

### Study population (undiagnosed diabetes)

**Figure 1:**
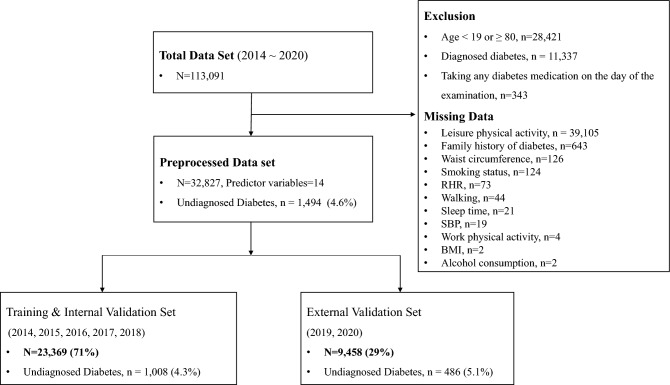
Flowchart of the study data set.

We used the data from the Korean National Health and Nutrition Examination Survey (KNHANES), which is an ongoing nationwide cross-sectional health and nutrition survey, to examine the health status of Koreans and to monitor trends in health risk factors and prevalence of major chronic diseases in Korea^15^. The details of the KNHANES have been described elsewhere^15^. Among individuals who participated in the 2014–2020 KNHANES (N = 113,091), we excluded those who were (1) aged < 19 or ≥ 80 years (N = 28,421); (2) diagnosed with diabetes (N = 11,337); and (3) missing data on predictor variables (N = 40,163; e.g., physical activity, family history of diabetes, WC, smoking status, RHR, sleep time, body mass index, alcohol consumption). Therefore, a total of 32,827 participants were examined. Figure 1 presents a flow chart of the study participants’ inclusion process.

Prediction algorithms and comparison between machine learning-based diabetes prediction model vs. traditional statistics-based diabetes prediction model.

We created ML-based prediction models based on the five ML classification algorithms: logistic regression, Random Forest^30^, Light Gradient Boosting Machine (Light GBM)^31^, Extreme Gradient Boosting (XG Boost)^32^, and Adaptive Boosting (AdaBoost)^33^. We compared the ML-based and TS-based prediction models using the previously developed diabetes prediction models^5,6^. These TS-based prediction models^5,6^ employed the previously established diabetes risk score^8^ and included easily accessible and publicly available clinical data from KNHANES, including sex, age, WC, family history of diabetes, hypertension status, smoking status, alcohol consumption, and/or RHR. Specifically, to compare ML-based prediction models and TS-based prediction models, we reproduced previous diabetes risk score models^5,6^ and compared their performance on the same external validation set. We compared ML-based prediction models and TS-based prediction models in four different sets of variables: (1) sex, age, WC, and RHR^6^; (2) sex, age, WC, hypertension status, alcohol consumption, smoking status, and family history of diabetes^5^; (3) sex, age, WC, hypertension status, alcohol consumption, smoking status, family history of diabetes, and RHR^6^; (4) in addition to the variables used in previous studies, features (i.e., predictor variables; e.g., physical activity, sleep time, and body mass index) that can maximize prediction performance were selected using the feature selection algorithm of machine learning. We utilized several methods of feature selection, including the Shapley value method^46^, the Recursive Feature Elimination Cross-Validation method^47^, and the Permutation feature selection method^48^. These approaches were employed to identify and include the main variables commonly selected across the different methods in our analysis. AUC was used to compare the prediction performance of the ML-based and the TS-based prediction models. We used the Hanley and McNeil’s methods^16^ to test the significant difference between the two AUC scores derived by the ML-based and TS-based prediction models.

### Measures

#### Target variable (undiagnosed type 2 diabetes)

Undiagnosed diabetes was previously defined and described^6^. Briefly, participants with fasting glucose ≥ 126 mg/dL or HbA1c ≥ 6.5% yet had not been diagnosed or under any diabetes treatments, were considered undiagnosed diabetes.

#### Features (predictor variables)

The methods of measurement have been previously described in detail. RHR was measured as a radial pulse in the right arm for 15 s after resting for 5 min in a seated position, and then multiplied by 4 and used as an RHR (beats/min). Age (years), family history of diabetes (yes, no), hypertension status (yes, no), smoking status (yes, no), and alcohol consumption (< 1, 1–4.9, 5 drinks/day) were measured using a general questionnaire administered by trained medical staff and interviewers. WC (cm) was measured at the narrowest point between the lower borders of the rib cage and the uppermost borders of the iliac crest at the end of a normal breath, using a standard protocol. The measurements of other features such as waist-to-height ratio (WHtR), body mass index (kg/m^2^), total physical activity (work-related, leisure-time, walking; metabolic equivalent task/week), and sleep time (h/day) are described in Supplemental Table 1.

**Table 1:** Participant characteristics according to data set.

	Training & internal validation set			External validation set		
	n = 23,369 (71.2%)			n = 9,458 (28.8%)		
	Non-diabetes	Undiagnosed diabetes	p-value	Non-diabetes	Undiagnosed diabetes	p-value
	n = 22,361	n = 1,008		n = 8,972	n = 486	
Age, yr	48.0 (15.8)	57.2 (12.3)*	< 0.001	47.9 (15.9)	57.2 (12.7)*	< 0.001
Height, cm	163.4 (9.1)	163.0 (9.5)	0.187	164.46 (9.2)	163.75 (9.3)	0.095
Weight, kg	63.5 (12.2)	70.0 (13.6)*	< 0.001	64.8 (13.0)	71.8 (15.3)*	< 0.001
BMI, kg/m^2^	23.7 (3.5)	26.2 (3.9)*	< 0.001	23.8 (3.6)	26.6 (4.2)*	< 0.001
WC, cm	80.7 (9.9)	89.0 (9.6)*	< 0.001	83.1 (10.3)	92.0 (10.2)*	< 0.001
WHtR	0.5 (0.1)	0.5 (0.1)*	< 0.001	0.5 (0.1)	0. 6 (0.1)*	< 0.001
RHR, bpm	69.4 (9.4)	72.1 (10.8)*	< 0.001	69.5 (9.6)	72.1 (10. 8)*	< 0.001
SBP, mmHg	116.8 (16.1)	126.3 (16.6)*	< 0.001	117.5 (15.8)	126.3 (15.6)*	< 0.001
DBP, mmHg	75.4 (10.0)	79.3 (10.9)*	< 0.001	76.0 (9.7)	79.7 (9.9)*	< 0.001
Sleep time, (hour/day)	7.1 (1.3)	7.0 (1.4)*	< 0.001	7.0 (1.3)	6.8 (1.3)*	< 0.001
Physical activity (MET-min/week)						
Work physical activity	55.8 (275.3)	62.73 (369.3)	0.441	96.6 (666.8)	64.16 (519.0)	0.291
Leisure physical activity	336.5 (818.2)	260.2 (738.8)*	0.004	326.9 (744.2)	239.9 (680.2)*	0.012
Walking	832.3 (1184.6)	844.3 (1366.2)	0.754	777.9 (938.6)	802.8 (998.1)	0.570
Total Physical activity	1714.3 (1985.7)	1643.3 (2317.2)	0.271	1614.3 (1803.3)	1603.1 (1861.0)	0.894
Sex			< 0.001			< 0.001
Men, n (%)	9,328 (41.7)	550 (54.6)		3,904 (43.5)	266 (54.7)	
Women, n (%)	13,033 (58.3)	458 (45.4)		5,068 (56.5)	220 (45.3)	
Family history of diabetes, n (%)	4,719 (21.1)	330 (32.7)	< 0.001	1978 (22.0)	179 (36.8)	< 0.001
Alcohol consumption (drinks/day),			< 0.001			< 0.001
< 1	17,996 (80.5)	738 (73.2)		7,318 (81.6)	366 (75.3)	
1–4.9	3,606 (16.1)	200 (19.8)		1,371 (15.3)	96 (19.8)	
≥ 5	759 (3.4)	70 (6.9)		283 (3.2)	24 (4.9)	
Smoking, n (%)	8,260 (19.5)	425 (23.3)	< 0.001	1,639 (17.4)	119 (23.2)	0.003
Hypertension, n (%)	14,720 (34.7)	1,025 (56.2)	< 0.001	2,492 (26.5)	275 (53.5)	< 0.001

Data are presented as mean (standard deviation) or number (%), All variables were tested by the T-test or chi-square test. Significant differences were found between non-diabetes, undiagnosed diabetes (p < 0.05), *Significantly different from non-diabetes. BMI = Body mass index, WC = Waist circumference, WHtR = Waist to Height Ratio, RHR = Resting heart rate, SBP = Systolic blood pressure, DBP = diastolic blood pressure, Total physical activity = Work physical activity + Leisure physical activity + Walking.

### Strategies for building diabetes prediction models

**Figure 2:**
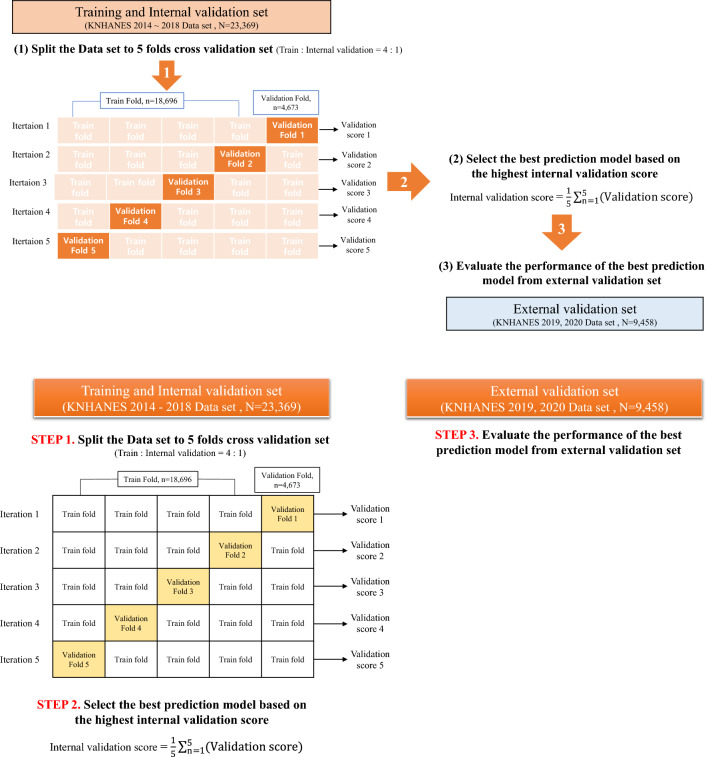
Conceptual schematic for prediction model building and performance evaluation.

Figure 2 shows the process of building a diabetes prediction model. We combined the KNHANES data from 2014 to 2020, and the 2014–2018 data (N = 23,369) were used as the training and internal validation sets and the 2019–2020 data (N = 9,458) as the external validation set. We then performed fivefold cross-validation using the training and internal validation sets to select an optimal prediction algorithm, hyper-parameters, and features, and to reduce the variance of the prediction performance (generated by the distribution of data when the data were randomly divided) to prevent overfitting of the model^17,18^. For the cross-validation, we used “Stratified Cross-Validation”^19^ after adjusting for the proportion of undiagnosed diabetes in each cross validation set. In the cross-validation process^20^, first, the prediction model was trained using the “Training set” and the performance of the trained model was examined using the “Internal validation set,” which was not included in the ‘Training set’. Second, we estimated the mean AUC values of the prediction performance level from the mutually exclusive 5 “Internal validation sets” after five iterations. Third, we selected the best prediction algorithm (when the estimated average of the AUC level was highest from the five internal validation sets), hyperparameters, and features for the prediction model. For reference, we utilized the Optuna framework^41^, which automates the search for the most effective hyperparameter configuration. Optuna offers a user-friendly and adaptable interface for defining search spaces, specifying the objective function for optimization, and choosing optimization algorithms^41^. Fourth, the highest mean AUC of the prediction model within the “Internal validation set” was validated using the 2019–2020 data (“external validation set”) and was compared with the TS-based Korean diabetes prediction models using risk scores^5,6^.

### Evaluation for the prediction performance of diabetes prediction models

We evaluated the performance of the diabetes prediction models using AUC, sensitivity, specificity, Youden index, positive predictive value (PPV), negative predictive value (NPR), positive likelihood ratio (PLR), and negative likelihood ratio (NLR). In general, the cutoff value of prediction models for predicting diabetes is determined when the Youden index (sensitivity + specificity − 1) is the highest^21^. However, considering the purpose of this study, we excluded the cutoff value of the highest Youden index (when sensitivity was low, and specificity was high) and determined the optimal cutoff value of the prediction model for diabetes when the sensitivity was greater than 80% and the specificity was greater than 50% (when the Youden index was highest).

### Shapely additive explanation (SHAP) analysis for interpretable ML models

Unlike traditional statistical methods, ensemble learning^22,23^, a type of ML algorithm (e.g., bagging and boosting) used in this study, is combined with multiple prediction models. Consequently, the prediction performance is superior to that of a single prediction model owing to the ensemble effect from combining multiple models^24^. However, it is difficult to clearly examine the features that contribute to prediction results^25^. To address this limitation, we adopted SHAP^26,27^, which is a leading unified framework for interpreting the decision-making process of ML models and prediction results^28,29^. For reference, SHAP analysis operates by assigning significance values, referred to as Shapley values (which represent the importance of each feature; positive values indicate a positive contribution), to the input features of a machine learning model. These values elucidate the extent to which each feature contributes to the model's prediction for a given instance^26,27^.

### Statistical analysis

We used Python version 3.8.8. to develop ML-based models and SPSS version 25.0 (Inc., Chicago, IL) for descriptive statistics, which includes frequency distributions and variability, were used to present the characteristics of the study participants. Differences between the non-diabetic and undiagnosed diabetes groups were examined using the t-test or chi-square test, as appropriate. The statistical difference of AUC between prediction models was examined using Hanley and McNeil’s methods^16^.

### Ethics approval and consent to participate

This study uses information disclosed to the public and was exempted from deliberation because it uses existing data that has already been generated information about the study subjects.

## Results

Participant characteristics stratified by data split (training & internal validation, and external validation sets) are shown in Table 1. Participants with undiagnosed diabetes (vs. non-diabetes) were more likely to be older and smokers, have higher body weight, body mass index, WC, RHR, WHtR, systolic and diastolic blood pressures, hypertension, more family history of diabetes, and greater alcohol consumptions (all P < 0.001) in both the “training & internal validation set” and “external validation set.” Furthermore, participants diagnosed with diabetes were more likely to be older and have a family history of diabetes and hypertension than participants with undiagnosed diabetes (all P < 0.05). For additional reference, the participant characteristics stratified by non-diabetes, undiagnosed diabetes, and diagnosed diabetes are presented in Supplemental Table 1.

The prediction performance comparison between the ML-based diabetes prediction model and TS-based prediction model^6^ using sex, age, WC, and RHR is presented in Table 2. In the external validation set, the AUC and Youden index of the TS-based prediction model developed by Park et al.^6^ were 0.740 (95% CI 0.721–0.759) and 35.0 respectively. Because the Random Forest showed the highest mean prediction performance in the training and internal validation sets, it was selected when four features (i.e., sex, age, WC, and RHR) were included in the model. In the external validation set, the AUC and Youden index of the ML-based prediction model were 0.788 (95% CI 0.722–0.804), 44.0, respectively. The AUC of the ML-based prediction model was significantly higher than that of the TS-based prediction model (P = 0.008).

**Table 2:** Performance of the new and Korean undiagnosed diabetes screening method in the development and validation datasets.

	Model	Screening method	Feature	AUC (95% CI)	Youden index	Sensitivity (%)	Specificity (%)	PPV	NPV	PLR	NLR
Train & Internal Validation Set	Park*	Risk score	Sex, Age, WC, RHR	0.745 (0.717 to 0.773)	37.00	70	66	0.08	0.98	2.09	0.45
	Logistic Regression	Logistic Regression		0.780 (0.754 to 0.806)	41.90	80.94	60.92	0.09	0.98	2.07	0.31
	Random Forest	Random Forest Classifier		0.781 (0.756 to 0.806)	41.20	84.60	56.60	0.08	0.99	2.1	0.16
	LGBM	LightGBM Classifier		0.778 (0.752 to 0.804)	41.70	82.00	61.60	0.08	0.99	2.14	0.29
	XGB	XGBoost Classifier		0.778 (0.752 to 0.804)	41.50	82.40	59.10	0.08	0.98	2.12	0.23
	Ada	AdaBoost Classifier		0.780 (0.754 to 0.806)	41.80	82.60	59.20	0.08	0.99	2.03	0.29
External Validation set	Park*	Risk score	Sex, Age, WC, RHR	0.740 (0.721 to 0.759)	35.00	75	61	0.09	0.98	1.9	0.42
	Logistic Regression	Logistic Regression		0.786 (0.77 to 0.802)	43.30	80.25	63.04	0.11	0.98	2.2	0.31
	Random Forest	Random Forest Classifier		0.788 (0.772 to 0.804)	44.00	87.40	56.50	0.18	0.99	2.01	0.22
	LGBM	LightGBM Classifier		0.788 (0.772 to 0.804)	43.70	82.90	60.80	0.1	0.99	2.12	0.28
	XGB	XGBoost Classifier		0.788 (0.772 to 0.804)	44.00	85.80	58.20	0.1	0.99	2.05	0.24
	Ada	AdaBoost Classifier		0.779 (0.762 to 0.796)	42.40	81.20	61.30	0.1	0.98	2.1	0.31

*Park et al. 2022^6^, When Park model’s performance was tested, data from 2019, 2020 were used to build prediction model and data from 2014, 2015, 2016, 2017, 2018 were used to validate. WC: Waist circumference, RHR: Resting heart rate, LGBM: Light Gradient Boosting Machine, XGB: Extreme Gradient Boosting), Ada: Ada Boost. AUC: The receiver operating characteristics curve under the curve. For this study, five different machine learning classification algorithms were used to predict undiagnosed diabetes. Based on their performance assessed by AUC, results from the best performed machine learning classification was used.

A comparison between the ML-based and TS-based diabetes prediction models^5^ using sex, age, WC, family history of diabetes, alcohol consumption, smoking status, and hypertension status is presented in Table 3. In an external validation set, the AUC and Youden index of the TS-based prediction model developed by Lee et al.^5^ were 0.759 (95% CI 0.741–0.777), and 36.0 respectively. Because XGBoost showed the highest mean prediction performance in the training and internal validation sets, XGBoost was selected when seven features (i.e., sex, age, WC, family history of diabetes, alcohol consumption, smoking status, and hypertension status) were included in the model. In the external validation set, the AUC and Youden index of the ML-based prediction model were 0.802 (95% CI 0.787–0.817), and 44.4 respectively. The AUC of the ML-based prediction model was significantly higher than that of the TS-based prediction model (P = 0.015).

**Table 3:** Performance of the new and Korean undiagnosed diabetes screening method in the development and validation datasets.

	Model	Screeing method	Feature	AUC (95% CI)	Youden index	Sensitivity (%)	Specificity (%)	PPV	NPV	PLR	NLR
Train & Internal Validation Set	Lee model*	Risk score	Sex, Age, WC, Family history of diabetes, Hypertension status, Smoking status, Alcohol consumption	0.750 (0.722 to 0.778)	36	86	51	0.07	0.99	1.74	0.28
	Logistic Regression	Logistic Regression		0.786 (0.761 to 0.811)	42.1	89.50	52.60	0.08	0.99	1.88	0.2
	Random Forest	Random Forest Classifier		0.781 (0.756 to 0.806)	43.5	82.70	60.80	0.08	0.98	2021	0.22
	LGBM	LightGBM Classifier		0.777 (0.751 to 0.803)	42.4	80.80	61.50	0.08	0.98	2.26	0.21
	XGB	XGBoost Classifier		0.786 (0.761 to 0.811)	42.7	82.80	61.20	0.08	0.98	2.31	0.18
	Ada	AdaBoost Classifier		0.785 (0.76 to 0.81)	42.4	80.30	62.10	0.08	0.99	2.12	0.32
External Validation set	Lee	Risk score	Sex, Age, WC, Family history of diabetes, Hypertension status, Smoking status, Alcohol consumption	0.759 (0.741 to 0.777)	36	90	46	0.08	0.99	1.67	0.21
	Logistic Regression	Logistic Regression		0.801 (0.786 to 0.816)	46.4	86.40	60.00	0.1	0.99	2.16	0.23
	Random Forest	Random Forest Classifier		0.792 (0.776 to 0.808)	46.1	83.00	63.10	0.11	0.99	2.25	0.27
	LGBM	LightGBM Classifier		0.795 (0.779 to 0.811)	45.8	81.90	64.00	0.11	0.98	2.27	0.28
	XGB	XGBoost Classifier		0.802 (0.787 to 0.817)	44.4	90.00	54.50	0.1	0.99	1.98	0.18
	Ada	AdaBoost Classifier		0.784 (0.768 to 0.8)	42.4	82.90	59.50	0.1	0.99	2.05	0.29

*Lee et al. 2012^5^, When Lee model’s performance was tested, data from 2019, 2020 were used to build prediction model and data from 2014, 2015, 2016, 2017, 2018 were used to validate. WC: Waist circumference, RHR: Resting heart rate, LGBM: Light Gradient Boosting Machine, XGB: Extreme Gradient Boosting, Ada: Ada Boost, AUC: The receiver operating characteristics curve under the curve.

For this study, five different machine learning classification algorithms were used to predict undiagnosed diabetes. Based on their performance assessed by AUC, results from the best performed machine learning classification was used.

A comparison between the ML-based diabetes prediction model and the TS-based prediction model^5^ using sex, age, WC, family history of diabetes, alcohol consumption, smoking status, hypertension status, and RHR is presented in Table 4. In the external validation set, the AUC and Youden index of the TS-based prediction model developed by Park et al.^6^ were 0.765 (95% CI 0.738–0.792) and 42.0 respectively. Since LightGBM showed the highest mean prediction performance in the training & internal validation sets, LightGBM was selected when eight features (i.e., sex, age, WC, family history of diabetes, alcohol consumption, smoking status, hypertension status, and RHR) were included in the model. In the external validation set, the AUC and Youden index of the ML-based prediction model were 0.811 (95% CI 0.796–0.826) and 48.3, respectively. The AUC of the ML-based prediction model was significantly higher than that of the TS-based prediction model (P = 0.008).

**Table 4:** Performance of the new and Korean undiagnosed diabetes screening method in the development and validation datasets.

	Model	Screening method	Feature	AUC (95% CI)	Youden index	Sensitivity (%)	Specificity (%)	PPV	NPV	PLR	NLR
Train and Internal validation set	Lee* + RHR	Risk score	Sex, Age, WC, RHR, Family history of diabetes, Hypertension status, Smoking status, Alcohol consumption	0.756 (0.728 to 784)	39	70	69	0.09	0.98	2.24	0.44
	Logistic Regression	Logistic Regression		0.799 (0.775 to 0.823)	45.4	83.20	62.20	0.09	0.99	2.21	0.27
	Random Forest	Random Forest Classifier		0.794 (0.77 to 0.818)	48.3	86.60	61.70	0.09	0.99	2.3	0.22
	LGBM	LightGBM Classifier		0.802 (0.778 to 0.826)	45.1	83.50	61.60	0.09	0.99	2.17	0.27
	XGB	XGBoost Classifier		0.796 (0.772 to 0.820)	44.9	81.40	63.50	0.09	0.99	2.35	0.23
	Ada	AdaBoost Classifier		0.796 (0.772 to 0.820)	44.3	80.80	63.50	0.09	0.99	2.21	0.3
External validation set	Lee* + RHR	Risk score	Sex, Age, WC, RHR, Family history of diabetes, Hypertension status, Smoking status, Alcohol consumption	0.765 (0.738 to 0.792)	42	78	64	0.11	0.98	2.17	0.35
	Logistic Regression	Logistic Regression		0.808 (0.793 to 0.823)	48.7	88.70	59.90	0.11	0.99	2.21	0.18
	Random Forest	Random Forest Classifier		0.807 (0.792 to 0.822)	47.6	83.50	64.03	0.11	0.98	2.32	0.26
	LGBM	LightGBM Classifier		0.811 (0.796 to 0.826)	48.3	84.00	64.30	0.11	0.99	2.35	0.25
	XGB	XGBoost Classifier		0.810 (0.975 to 0.825)	48	85.20	63.00	0.11	0.99	2.29	0.23
	Ada	AdaBoost Classifier		0.800 (0.784 to 0.816)	46.3	84.50	61.80	0.11	0.99	2.21	0.25

*Lee et al. 2012^5^ and Park et al. 2022^6^ When Lee model’s + RHR (Park et al., 2022) performance was tested, data from 2019, 2020 were used to build prediction model and data from 2014, 2015, 2016, 2017, 2018 were used to validate. WC: Waist circumference, RHR: Resting heart rate, LGBM: Light Gradient Boosting Machine, XGB: Extreme Gradient Boosting, Ada: Ada Boost. AUC: The receiver operating characteristics curve under the curve. For this study, five different machine learning classification algorithms were used to predict undiagnosed diabetes. Based on their performance assessed by AUC, results from the best performed machine learning classification was used.

In addition to the aforementioned features from previous TS-based diabetes prediction models^5,6^, the feature selection algorithm determined a total of 11 features (previous features plus four additional features: body mass index, WHtR (replacement of WC), physical activity, and sleep time). A comparison between the ML-based diabetes prediction model and TS-based diabetes prediction models^5,6^ using these 11 features is presented in Table 5. In the external validation set, LightGBM (the highest prediction performance in the training & internal validation sets) showed the highest prediction performance. The AUC and Youden index of this ML-based prediction model were 0.819 (95% CI 0.805–0.833) and 47.4, respectively. The AUC of the ML-based prediction model was significantly higher than that of the TS-based prediction model (P = 0.001).

**Table 5:** Performance of the new and Korean undiagnosed diabetes screening method in the development and validation datasets.

	Model	Screening method	Feature	AUC (95% CI)	Youden index	Sensitivity (%)	Specificity (%)	PPV	NPV	PLR	NLR
Train and Internal validation set	Lee* + RHR	Risk score	Sex, Age, WC, RHR, BMI, Family history of diabetes, Hypertension status, Smoking status, Alcohol consumption, Physical activity, Sleep time	0.756 (0.728 to 0.784)	39	70	69	0.09	0.98	2.24	0.44
	Logistic Regression	Logistic Regression		0.801 (0.777 to 0.825)	43.6	80.50	63.10	0.08	0.99	2.2	0.31
	Random Forest	Random Forest Classifier		0.788 (0.763 to 0.813)	44.8	82.30	62.40	0.09	0.98	2.35	0.19
	LGBM	LightGBM Classifier		0.803 (0.779 to 0.827)	45.9	80.70	65.20	0.09	0.99	2.58	0.17
	XGB	XGBoost Classifier		0.797 (0.773 to 0.821)	44.7	81.70	63.00	0.09	0.98	2.41	0.18
	Ada	AdaBoost Classifier		0.786 (0.761 to 0.811)	43.7	82.50	61.20	0.08	0.98	2.31	0.18
External validation set	Lee + RHR	Risk score	Sex, Age, WC, RHR, BMI, Family history of diabetes, Hypertension status, Smoking status, Alcohol consumption, Physical activity, Sleep time	0.765 (0.748 to 0.782)	42	78	64	0.11	0.98	2.17	0.35
	Logistic Regression	Logistic Regression		0.814 (0.799 to 0.829)	47.4	87.40	60.00	0.11	0.99	2.2	0.21
	Random Forest	Random Forest Classifier		0.815 (0.8 to 0.83)	48.7	88.70	60.00	0.1	0.99	2.2	0.19
	LGBM	LightGBM Classifier		0.819 (0.805 to 0.833)	49.6	84.80	64.80	0.11	0.99	2.41	0.23
	XGB	XGBoost Classifier		0.818 (0.804 to 0.832)	49.5	82.90	66.60	0.11	0.98	2.48	0.25
	Ada	AdaBoost Classifier		0.809 (0.786 to 0.816)	46.5	83.90	62.50	0.11	0.98	2.24	0.26

*Lee et al. 2012^9^ and Park et al. 2022^10^ When Lee model’s + RHR (Park et al., 2022) performance was tested, data from 2019, 2020 were used to build prediction model and data from 2014, 2015, 2016, 2017, 2018 were used to validate. WC: Waist circumference, RHR: Resting heart rate, LGBM: Light Gradient Boosting Machine, XGB: Extreme Gradient Boosting, Ada: Ada Boost, AUC: The receiver operating characteristics curve under the curve. For this study, five different machine learning classification algorithms were used to predict undiagnosed diabetes. Based on their performance assessed by AUC, results from the best performed machine learning classification was used.

**Figure 3:**
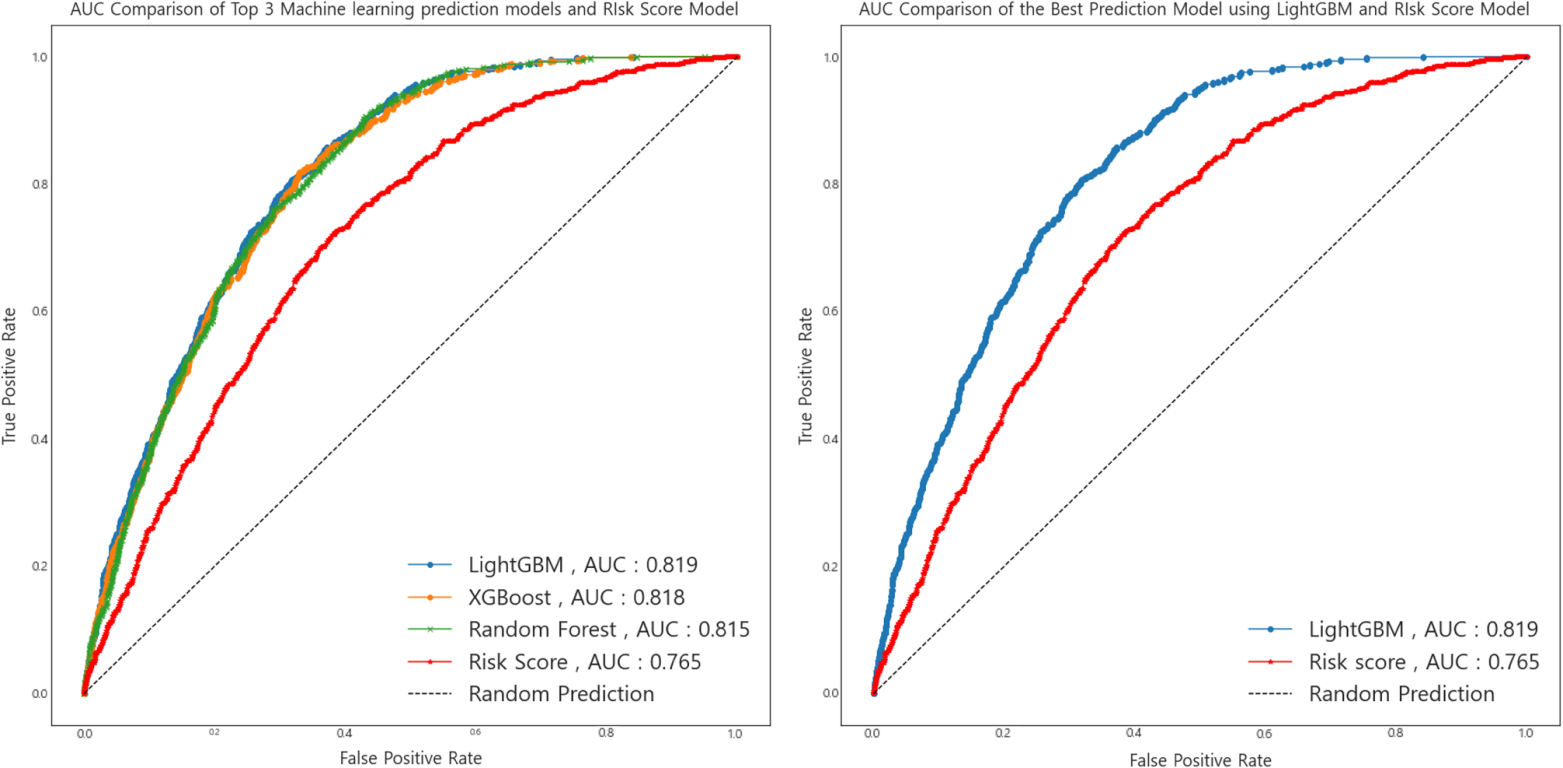
AUC Comparison of machine learning prediction models and risk score model.

Figure 3 shows the highest 3 AUC of the ML-based diabetes prediction models and the model with the highest AUC among the previous TS-based diabetes prediction models developed by Park et al. ^6^ .

**Figure 4:**
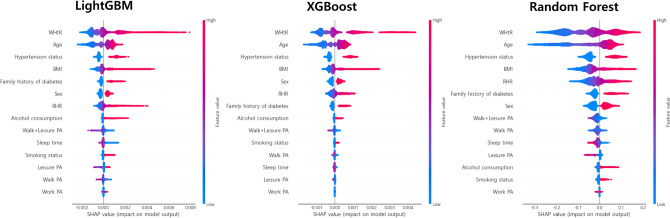
SHAP summary plot of the top 3 prediction models: contribution and effect of each feature.

After validating the prediction performance, we used SHAP framework^26,27^. Figure 4 shows the SHAP summary results of the top three machine-learning-based models. The SHAP values differed slightly among the prediction algorithms. The WHtR, age, hypertension status, body mass index, family history of diabetes, sex, and RHR were selected as important features with a high contribution to the detection of undiagnosed diabetes. According to the SHAP value, as the WHtR, age, body mass index, and RHR values increased, the probability that the prediction model predicted the participant to have diabetes increased. The contribution of lifestyle features (e.g., alcohol consumption, physical activity, sleep time, and smoking status) to the prediction results was relatively small compared with the anthropometric measures (e.g., WHtR, age, body mass index, and RHR). The lower the levels of physical activity and sleep time and the higher the work physical activity, the higher the probability of being diagnosed with undiagnosed diabetes. In the case of categorical features, the probability of predicting undiagnosed diabetes using the prediction model was higher in male, having a family history of diabetes, hypertension, current smoking, and high alcohol consumption.

## Discussion

We compared the prediction performance of the ML-based prediction models with that of the TS-based diabetes prediction models with an external validation set in a large representative sample of Korean adults, using self-reported clinical data. Our findings suggest that ML-based diabetes prediction models, regardless of the number of features used in developing models, were superior to TS-based prediction models^5,6^ using the diabetes risk score method^8^. When the feature selection method was employed in our ML-based model, the AUC was 0.819, which was better than the highest AUC (0.765) among TS-based models^6^.

Some assumptions explain why the ML-based diabetes prediction models used in this study were superior to the TS-based prediction models. First, the ML methods we used in our study were bagging^34^ and boosting^35^ algorithms^22^, which developed multiple prediction models, aggregated to determine the final prediction result. Since the final prediction result is determined by voting for various prediction results, an unbiased prediction result can be obtained^23,36^. Compared with a single prediction model, these methods result in a more accurate prediction^34–39^. Second, when compared to the ML-based approach, the TS-based approach^5,6,8^ is challenging for researchers to develop prediction models by considering all possible cases that may result from multiple features and algorithms. In contrast, an ML-based method can select the optimal features to maximize the prediction performance using feature selection algorithms^40^. In addition, by using hyperparameter tuners such as Optuna^41^ and Hyperopt^42^, it is possible to determine how many single prediction models are combined to develop a final prediction model to maximize prediction performance while avoiding overfitting. Our findings suggest that diabetes prediction models developed by the ML-based method may be more time-efficient, cost-effective, and superior to the previous TS-based method.

For these reasons, there is growing evidence for the application of the ML-based approach and artificial neural network, a type of ML, to develop prediction models for diabetes^11,12,14,43,44^. However, these prediction models^11,12,43^ may be less accessible because they were developed using blood lipid variables (e.g., fasting glucose, HbA1c, triglyceride, and total cholesterol). In addition, another study^14^ using XGBoost, an algorithm similar to our approach, reported a high AUC score of 0.92. However, this prediction model^14^ may be at high risk of overfitting^45^ given that the prediction model was developed without using the ‘external validation set’. In addition, the prediction performance of this model^14^ was not assured, given that there was no verified result for unseen data. On the other hand, our ML-based prediction model developed using non-invasive data may be more accessible. Furthermore, the external validity of our prediction model was tested from the external validation set and we used the SHAP analysis to determine the predictive power of each predictor (feature) and to generate explainable models, while the previous artificial neural network prediction model for undiagnosed diabetes^44^, deemed a black-box model, using non-invasive data (e.g., age, WC, body mass index, sex, smoking status, hypertension, and family history of diabetes) did not validate their model through the application of SHAP analysis.

In addition, the aforementioned prediction models only mentioned the prediction performance and did not explain the importance or effect of the features that contributed to the prediction results. Therefore, it was impossible to interpret the prediction models used in these studies. To address this limitation, the ML-based prediction model of this study calculated the contribution and effect of each feature using SHAP and presented it to interpret its prediction results. Additionally, the sensitivity of our prediction model using age, WC, and RHR was 83.3%, which may be sufficiently valid.

This study has several limitations. First, given the nature of the cross-sectional study design, we could not determine causality between the features and undiagnosed diabetes. Thus, future studies on diabetes prediction models should employ longitudinal cohort data to examine the temporal relationships between features and incident diabetes. Additionally, RHR is highly affected by sleep quality, smoking status, alcohol consumption, and/or major health characteristics; therefore, interpretation should be made with caution. Lastly, findings cannot be generalized to wider populations given that our study examined Korean data only. Thus, additional research with racially/ethnically diverse population data is needed to confirm our preliminary findings.

In conclusion, our study suggests that ML-based undiagnosed type 2 diabetes prediction models may improve the prediction performance of TS-based prediction models and methods. The continuous increase in the number of diagnosed and undiagnosed diabetes epidemics is a major public health concern. The study findings will inform public health researchers and healthcare professionals to apply efficient new diabetes prediction models for the prevention of diabetes and its adverse health consequences. A clear next step in future research is to identify our preliminary findings in a different setting of data with wider populations in order to better generalize the findings.

### Supplementary Information

Supplementary Information.

## Data Availability

All data generated or analyzed during this study are included in this published article and are available from the Korean National Health & Nutrition Examination Survey repositories.

## Abbreviations

WC: Waist circumference
WHtR: Waist to height ratio
RHR: Resting heart rate
DRS: Diabetes risk score
KNHANES: Korean National Health and Nutrition Examination Survey
ROC: Receiver operating characteristic
AUC: Area under the ROC curve
ML: Machine learning
TS: Traditional statistics
PPV: Positive predictive value
NPV: Negative predictive value
PLR: Positive likelihood ratio
NLR: Negative likelihood ratio
SHAP: Shapely additive explanation
LightGBM: Light gradient boosting machine
XGBoost: Extreme gradient boosting machine
AdaBoost: Adaptive boosting
Bagging: Bootstrapping and aggregating
