# Supplementary material for: Comparisons of the prediction models for undiagnosed diabetes between machine learning versus traditional statistical methods
Source: Sci Rep. 2023 Aug 11;13:13101. doi: 10.1038/s41598-023-40170-0 (PMC10421881; doi:10.1038/s41598-023-40170-0)
Supplement: Supplementary file 1 — Supplementary Information. [file 41598_2023_40170_MOESM1_ESM.docx]

**Comparisons of the prediction models for undiagnosed diabetes between machine learning vs. traditional statistical methods**

Seong Gyu Choi^1^†, Minsuk Oh^1, 2^†, Dong–Hyuk Park^1^, Byeongchan Lee^3^, Yong-ho Lee^4^, Sun Ha Jee^5^, Justin Y. Jeon^1, 2, 6, 7*^.

¹Department of Sports industry, Yonsei University, Seoul, Korea; ^2^Frontier Research Institute of Convergence Sports Science, Yonsei University, Seoul, Korea; ^3^Gauss Labs, Seoul, Korea; ^4^Department of Internal Medicine, Yonsei University College of Medicine, Seoul, Korea; ^5^Institute for Health Promotion, Graduate School of Public Health, Yonsei University, Seoul, Korea; ^6^Exercise Medicine Center for Diabetes and Cancer Patients, ICONS; ^7^Cancer Prevention Center, Shinchon Severance, Yonsei University College of Medicine, Seoul Korea

**Supplemental Table 1**. Measurements for other features (predictors)

| **Features** | **Measurement description** |
| --- | --- |
| Body mass index (kg/m^2^) | Height (to the nearest 0.1 cm) and body weight (to the nearest 0.1 kg) were measured using a height-measuring device (Seca 225; GmbH&Co. KG, Hamburg, Germany) and a portable digital scale (GL-6000-20; Caskorea, Seoul, South Korea), respectively. Body mass index was calculated as weight (kg) divided by height squared (m^2^). |
| Waist circumference (cm) | Waist circumference (cm) was measured at the point between the lowest rib and the top of the iliac crest using an ergonomic circumference measuring tape (Seca 201; GmbH&Co.KG). |
| Waist-to-height ratio (%) | Waist-to-height ratio was calculated as waist (cm) divided by height (cm). |
| Blood pressure (mmHg) | Blood pressure was measured on the right arm with the participant in a seated position after a 5 min rest period using a standard digital blood pressure monitor (Microlife WatchBP Office AFIB). Three measurements were recorded at 5 min intervals and an average of the last two measurements was used for analysis. |
| Physical activity (MET-min/week) | Leisure-time moderate and vigorous-intensity physical activities, work-related physical activities, and total time in walking (min/week) were assessed in the KNHANES health interview using the Korean version of the modified Global Physical Activity Questionnaire (GPAQ) [1]. The GPAQ assessed the frequency (days/week) and duration (min/day) spent in physical activities in bouts of at least 10 minutes duration. Based on the International Physical Activity Questionnaire scoring protocol [3], the score with the unit of metabolic equivalent (MET)-min/week was estimated by multiplying the MET score of each intensity (8.0 for vigorous, 4.0 for moderate, and 3.3 for walking) by total min/day and the number of days of participating [1-4]. |
| Sleep time (hour/day) | The total sleep was calculated in minutes by having the subjects record the time they went to bed and woke up on weekdays and weekends, and the average value was used as the average sleep time per day (hour/day). |

**References**

1. Lee J, Lee C, Min J, et al. Development of the Korean global physical activity questionnaire: reliability and validity study. *Glob Health Promot*. 2019;27(3):44–55. Epub 2019 Aug 2.

2. Ainsworth, B. E., Haskell, W. L., Whitt, M. C. et al. Compendium of physical activities: an update of activity codes and MET intensities. *Med Sci Sports Exerc*. 2000;32(9; SUPP/1), S498-S504.

3. IPAQ Research Committee. International physical activity questionnaire. https://sites.google.com/site/theipaq/. Accessed October 6, 2022.

4. Park J, Shin A, Lee M, et al. Prevalence of participating in physical activity from 2 Korean surveillance systems: KNHANES and KCHS. *J Phys Act Health*. 2018;15(10):763-773

| **Supplemental Table 2. Characteristics of participants** | | | | |
| --- | --- | --- | --- | --- |
|  | **KNHANES 2010 ~ 2020 Data set** | | | |
|  | n=35,988 | | | |
|  | **Non-diabetes** | **Undiagnosed diabetes** | **Diagnosed diabetes** | p-value |
|  | n=31,333 | n=1,494 | n=3,161 |  |
| Age, yr | 47.95±15.78 | 57.19±12.37* | 63.6±10.50*# | <0.001 |
| Height, cm | 163.7±9.13 | 163.2±9.46 | 161.3±8.92*# | <0.001 |
| Weight, kg | 63.85±12.43 | 70.56±14.17* | 65.72±11.79*# | <0.001 |
| BMI, kg/㎡ | 23.72±3.50 | 26.34±3.99* | 25.15±3.46*# | <0.001 |
| WC, cm | 81.69±9.87 | 90.24±9.55* | 88.54±9.17*# | <0.001 |
| WHtR | 0.49±0.06 | 0.55±0.05* | 0.50±0.06*# | <0.001 |
| RHR, bpm | 69.48±9.40 | 71.11±10.77* | 71.15±10.32* | <0.001 |
| SBP, mmHg | 117.0±16.0 | 126.5**±**16.3* | 125.3±15.9*# | <0.001 |
| DBP, mmHg | 75.59±9.9 | 79.43±10.6* | 73.43±9.73*# | <0.001 |
| Sleep time, (hour/day) | 7.09±1.32 | 6.90±1.41* | 6.90±1.51* | <0.001 |
| Physical activity (METs/week) |  |  |  |  |
| Work Physical activity | 67.47±426.3 | 63.19±423.7 | 43.45±350.6* | 0.009 |
| Leisure Physical activity | 333.8±797.7 | 253.6±720.1* | 226.5±749.6* | <0.001 |
| Walk Physical activity | 816.7±1120 | 830.8±1258 | 857.4±1267 | 0.151 |
| Total Physical activity | 1685±1935 | 1630±2178 | 1526±1916* | <0.001 |
| **Sex** |  |  |  | <0.001 |
| Men, n (%) | 13,232 (42.2) | 816 (54.6) | 1,608 (50.9) |  |
| Women, n (%) | 18,101 (57.8) | 678 (45.4) | 1,553 (49.1) |  |
| Family history of diabetes, n (%) | 6,697 (21.4) | 509 (34.1) | 1,385 (43.8) | <0.001 |
| Alcohol intake (drinks/day), n (%) |  |  |  | <0.001 |
| <1 | 25,314 (80.8) | 1,104 (73.9) | 2,572 (81.4) |  |
| 1-4.9 | 4,977 (15.9) | 296 (19.8) | 440 (13.9) |  |
| ≥5 | 1,042 (3.3) | 94 (6.3) | 149 (4.7) |  |
| Smoking, n (%) | 5,636 (18.0) | 350 (23.4) | 579 (18.3) | <0.001 |
| Hypertension, n (%) | 8,132 (26.0) | 813 (54.4) | 2,085 (66.0) | <0.001 |
| Data were presented as mean ± standard deviation or number (%), All variables were tested by ANOVA or Chi-square test. Significant differences were found between non-diabetes, undiagnosed diabetes, Diagnosed diabetes (p<0.05), *Significantly different from non-diabetes, #Significantly different from undiagnosed diabetes BMI=Body mass index, WC=Waist circumference, WHtR=Waist to Height Ratio, RHR=Resting heart rate, SBP=Systolic blood pressure, DBP=diastolic blood pressure, Total physical activity = Work physical activity + Leisure physical activity + Walking | | | | |

|  |  |
| --- | --- |
|  |  |
